# Endoscopic full-thickness resection with defect closure using an over-the-scope clip for gastric subepithelial tumors originating from the muscularis propria

**DOI:** 10.1007/s00464-015-4076-2

**Published:** 2015-02-21

**Authors:** Jintao Guo, Zhijun Liu, Siyu Sun, Xiang Liu, Sheng Wang, Nan Ge, Guoxin Wang, Yafei Qi

**Affiliations:** 1 Endoscopic Center, Shengjing Hospital of China Medical University, No. 36 Sanhao Street, Shenyang, 110004 Liaoning Province China; 2 Ultrasound Department, Shengjing Hospital of China Medical University, No. 36 Sanhao Street, Shenyang, 110004 Liaoning Province China; 3 Pathological Department, Shengjing Hospital of China Medical University, No. 36 Sanhao Street, Shenyang, 110004 Liaoning Province China

**Keywords:** OTSC, Stomach, Submucosal tumor, Muscularis propria, Full-thickness resection

## Abstract

**Background:**

Endoscopic full-thickness resection (EFTR) is a mini-invasive technique for gastric subepithelial tumors originating from the muscularis propria, which enables a full-thickness resection of tumors and can provide a complete basis for pathological diagnosis. Gastric fistula closure after EFTR is a challenge for endoscopists. In this study, we introduced EFTR with fistula closure using the over-the-scope clip (OTSC) system for gastric subepithelial tumors originating from the muscularis propria.

**Objectives:**

To evaluate the feasibility and safety of fistula closure with OTSC by a retrospective analysis on the cases of EFTR with defect closure using OTSC for gastric subepithelial tumors originating from the muscularis propria in our hospital.

**Methods:**

The patients were selected who underwent EFTR for gastric subepithelial tumors originating from the muscularis propria (tumor diameter ≤2 cm) in our hospital from October 2013 to March 2014. After a full-thickness resection of tumors, the bilateral gastric mucous membranes of defect were clamped using twin graspers and then drawn into the transparent cap of OTSC, and the OTSC was released to close the defect after full suctioning. The success rate of defect closure with OTSC was observed, and the endoscopic follow-up was performed at 1 week, 1 and 6 months after operation to check OTSC closure.

**Results:**

Totally 23 patients were included into the study. The full-thickness resection rate of gastric tumors in the muscularis propria was 100 % (23/23), the success rate of defect closure was 100 %, and the average time of defect closure was 4.9 min (range 2–12 min). All patients experienced no postoperative complications such as bleeding and perforation. The postoperative follow-up time was 1–6 months (mean 3 months), and no OTSC detachment was found.

**Conclusions:**

OTSC can be used to perform EFTR with defect closure for gastric tumors in the muscularis propria (tumor diameter ≤2 cm). It is simple, convenient, safe and effective.

**Electronic supplementary material:**

The online version of this article (doi:10.1007/s00464-015-4076-2) contains supplementary material, which is available to authorized users.

A gastrointestinal stromal tumor originating from the muscularis propria can be malignant; therefore, a reliable, full-thickness tumor resection is required. Considering the above, surgical and laparoscopic procedures are currently important techniques to treat larger gastric stromal tumors. For small, asymptomatic gastric tumors in the muscularis propria, however, it remains controversial whether to choose resection or regular follow-up [1]. In addition, this difficult decision is usually made by the patient.

In recent years, endoscopic dissection or an enucleation technique has been used for the resection of gastrointestinal tumors in the muscularis propria; however, gross resection (R1 resection) is often successfully performed under endoscopy in most cases; complete resection (R0 resection) can be achieved only in a few cases. Endoscopic full-thickness tumor resection (and obtaining peri-tumor normal gastric tissues) could be a more minimally invasive choice for patients, provided that it could achieve the same therapeutic effect as a laparoscopic procedure for submucosal tumors. However, closing gastric defects quickly and reliably after full-thickness tumor resection is a challenge for endoscopists. In the present study, we proposed a new method to close gastric defects after a full-thickness gastric resection, i.e., defect closure with the over-the-scope clip (OTSC).

The OTSC closure system (Ovesco Endoscopy GmbH, Tuebingen, Germany) has been successfully applied to the treatment of gastrointestinal bleeding, fistulas and perforations [2]. For resection of gastrointestinal submucosal tumors not originating from the muscularis propria and wound closure, the feasibility of the OTSC closure system has been proven [3]. However, the application of the OTSC closure system in endoscopic full-thickness resection (EFTR) with gastric fistula closure for gastric tumors in the muscularis propria has been rarely described. Previous studies have reported that the metal clip was often used to close gastric fistula after EFTR [4].

In order to evaluate the safety and efficacy EFTR, we retrospectively analyzed the cases that underwent the procedure with defect closure using OTSC for gastric tumors in the muscularis propria.

## Patients and methods

### Patient data

We retrospectively analyzed the patients who underwent EFTR for gastric tumors in the muscularis propria (tumor diameter ≤2 cm) in our hospital from October 2013 through March 2014. Before EFTR, all patients received computed tomography (CT) and endoscopic ultrasound (EUS) examinations to determine the originating layer of the gastric tumors, their relationship with adjacent great vessels of the stomach and the presence/absence of peripheral lymph node enlargement; thus, we excluded all malignant diseases. If a tumor originated from the muscularis propria (tumor diameter ≤2 cm) and there was no peripheral lymph node enlargement and metastasis, EFTR was performed. All patients were hospitalized for treatment.

This study was approved by the Institutional Review Board and Ethics Committee of China Medical University. All patients voluntarily chose their therapeutic course and provided written informed consent for their participation in this study. The operator performing the EFTR procedure in this study was familiar with both endoscopic submucosal dissection and EFTR techniques.

### Endoscopic equipment and accessories

A standard single-channel gastroscope (EPK-i, Pentax) was used throughout the endoscopic procedure; a linear array ultrasonic endoscope (EG3830UR; Pentax Precision Instrument, Orangeburg, New York) was used for the evaluation of tumor size, echo characteristics and the originating layer. A triangle-tipped knife and insulated-tip knife (both from Olympus Corporation, Tokyo, Japan) was used for the dissection and resection of tumors, a hot biopsy forceps (FD-410LR) was used for gastric wall hemostasis, twin graspers (Ovesco Endoscopy GmbH, Tuebingen, Germany) were used for clamping the two sides of the gastric defect, and metal clips (Olympus Corporation, Tokyo, Japan) and the OTSC system (Ovesco Endoscopy GmbH, Tuebingen, Germany) were used for defect closure.

### Procedure

1. EFTR

**Fig. 1:**
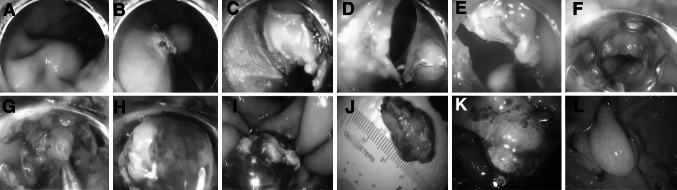
**A** A protruding tumor with a smooth surface in the gastric fundus **B** Peri-tumor gastric mucosa was incised **C** Peri-tumor gastric submucosa was dissected **D**, **E** Full-thickness resection was performed around the tumor **F** Gastric defect formed after full-thickness tumor resection. The gastric walls at both margins of the defect were clamped with twin graspers (**G**) and drawn into the transparent cap of the OTSC for full suction (**H**) **I** OTSC clamping device was released to approximate the gastric walls at the defect **J** The full-thickness structure of the peri-tumor gastric wall is observed **K** Gastroscopic reexamination 1 week postoperatively revealed edema of the gastric wall **L** Gastroscopic reexamination 6 months postoperatively revealed a nearly normal gastric wall

The peri-tumor gastric tissues were incised with a triangle-tipped knife, and then, the tumor and peri-tumor gastric tissues were gradually resected in full thickness using an insulated-tip knife (with the aim of protecting adjacent organs beneath the gastric wall) (Fig. 1a–e). An iatrogenic gastric perforation was created (Fig. 1f). During the resection, hemostasis was obtained with electric cautery.

2. Wound closure with the OTSC system

Gastric tissues adjacent to the iatrogenic perforation were clamped with twin graspers and then drawn into the transparent cap of the OTSC device; then, they were fully aspirated into the transparent cap, and the OTSC closure system was released to close the defect (Fig. 1g–i). If defect closure was incomplete, metal clips were used to close the remaining portions. After the gastric defect was completely closed, gastric gas was fully aspirated and the gastroscope was withdrawn.

All EFTR and OTSC closure system operations were performed by one experienced endoscopist. Carbon dioxide was injected throughout the procedure. A 20-ml syringe was used to aspirate free gas from the abdomen during or after the procedure. A gastric decompression tube, which could also observe postoperative bleeding, was indwelled into the stomach. The postoperative treatment included 24 h of fasting (both food and water) as well as routine administration of proton pump inhibitors (PPIs) and antibiotics. All patients were closely observed. If significant hemorrhage occurred, hemostasis was obtained endoscopically or surgically. If peritonitis symptoms were observed, the indwelling time of the gastric decompression tube and the duration of antibiotic administration were usually increased. If conservative treatment was unsuccessful, surgical treatment was performed. On postoperative day 2, the patients without postoperative bleeding and peritonitis received a liquid diet and oral PPIs. If the patients did not experience discomfort and had normal laboratory studies after resuming their diet, they continued taking oral PPIs for 1 month after discharge.

At 1 week, 1 and 6 months postoperatively, endoscopic follow-up was performed to assess the wound and defect closure (Fig. 1k and l).

### Statistical analysis

Statistical analysis was performed with the SPSS software package version 19.0 (SPSS Inc., Chicago, IL, USA).

## Results

This study comprised 23 patients aged 54.8 ± 9.7 (range 32–70) years, including 13 males (57 %) and 10 females (44 %). The mean tumor diameter was 12.1 ± 4.7 (range 6–20) mm. The tumor was located in the gastric antrum in three cases, in the gastric body in nine cases, and in the gastric fundus in 11 cases. The full-thickness resection rate of gastric tumors in the muscularis propria was 23/23 (100 %), and the EFTR time was 40.5 ± 25.8 (range 16–104) min. The rate of no residual tumor at the cutting edge was 23/23 (100 %) as documented by pathological diagnosis. Nineteen cases (19/23; 83 %) were stromal tumors, in which one case (1/19; 5 %) was at high risk and the remaining cases (18/19; 95 %) were at very low risk; four cases (4/23; 17 %) were leiomyomas. The success rate of defect closure was 100 %. In 23 patients, complete defect closure was achieved with only one OTSC. The defect closure time was 4.9 ± 2.2 (range 2–12) min. The incidence of fever (*T* > 37 °C) was 3/23 (13 %) on the day of operation, 1/23 (4 %), on postoperative day 1, and 0/23 (0 %) on postoperative day 2. Two patients (9 %) experienced localized peritonitis postoperatively and improved following conservative treatment. None of the patients developed postoperative bleeding or perforation. The mean postoperative hospital stay was 3 (range 2–5) days. The postoperative follow-up time was 3 (range 1–6) months, and no patient had OTSC detachment during follow-up. Patient demographics, lesion features, pathological diagnosis and clinical outcomes are summarized in Table 1.

**Table 1:** Demographic and clinicopathological characteristics of the study patients (*N* = 23)

Case no.	Gender/age (years)	Location	Tumor size (diameter, mm)	Defect closure time (min)	Operation time (min)	Pathological diagnosis
1	M/60	Antrum	20	12	104	GIST
2	F/46	Body	20	7	87	GIST
3	F/32	Body	17	6	63	GIST
4	M/38	Fundus	6	6	39	Leiomyoma
5	F/42	Fundus	12	8	42	GIST
6	M/69	Body	11	3	35	GIST
7	M/63	Body	14	4	31	GIST
8	M/66	Fundus	8	4	19	Leiomyoma
9	M/47	Fundus	8	6	26	GIST
10	F/52	Antrum	16	3	103	GIST
11	M/54	Body	20	4	47	GIST
12	F/57	Body	6	4	17	GIST
13	M/59	Body	13	7	27	GIST
14	M/65	Body	7	3	22	GIST
15	M/62	Fundus	6	3	31	GIST
16	M/50	Body	12	4	29	GIST
17	F/54	Fundus	8	2	16	GIST
18	M/70	Antrum	14	6	36	Leiomyoma
19	F/49	Fundus	10	5	25	GIST
20	F/56	Fundus	14	5	31	GIST
21	F/51	Fundus	7	4	18	Leiomyoma
22	M/60	Fundus	17	3	56	GIST
23	F/58	Fundus	12	3	28	GIST

## Discussion

There are various types of gastric tumors originating from the muscularis propria, among which a potential malignant tumor, i.e., gastrointestinal stromal tumor, is the most common type [5, 6]. Resection is recommended for gastric tumors in the muscularis propria that are large and/or symptomatic; however, either long-term endoscopic follow-up or resection may be selected for those that are small and asymptomatic [1]. A disadvantage of long-term endoscopic follow-up is its burden on the patient. Currently, the imaging procedures (CT and EUS) cannot accurately determine the pathological diagnosis of submucosal tumors, and it is difficult to biopsy small tumors under EUS [7, 8]. The patients often express concern regarding tumor growth; thus, they are impacted with a psychological burden. Moreover, the tumors suspected to be gastric stromal tumors have the risk of growth and malignant transformation. Based on the above reasons, if a minimally invasive technique is available to resect even small gastric tumors in the muscularis propria, most patients prefer resection over long-term endoscopic follow-up.

Studies have demonstrated that endoscopic submucosal dissection (ESD) and endoscopic submucosal enucleation (ESE) are feasible for the resection of gastric tumors in the muscularis propria [9, 10]. However, it is difficult to achieve R0 resection (resection of tumor and peri-tumor normal gastric tissues) by endoscopic dissection; thus, the risk of recurrence exists. Of course, laparoscopic surgery can attain R0 resection; however, it is inferior for the detection of small tumors and is also more invasive than endoscopic resection. In recent years, EFTR has been used for full-thickness resection of gastric tumors in the muscularis propria [11–14]. EFTR enables full-thickness resection of tumors; thus, it provides for attainment of a complete tumor specimen for pathologic study. EFTR facilitates the determination of whether residual tumor is present at the resection margins and also determines whether there is a need for subsequent treatment; thus, it reduces the possibility of residual tumor and recurrence. Shi et al. [11] successfully performed 20 cases of EFTR for gastric tumors in the muscularis propria; they reported a full-thickness resection rate of 100 % and diameters of resected tumors of 1.47 ± 0.72 cm. Zhou et al. [12] resected gastric tumors originating from the muscularis propria in 26 cases by EFTR, with a full-thickness resection rate of 100 % and an average surgery time of 105 min. Dong et al. [15] compared the efficacy of laparoscopic resection vs. EFTR for gastric tumors originating from the muscularis propria and concluded that both approaches achieved R0 resection; furthermore, the size of the resected tumors differed insignificantly between them.

It is technically challenging for endoscopists to close a gastric fistula after EFTR. With continuous improvements in endoscopic adjunctive therapeutic equipment, additional methods for gastric defect closure are emerging; OTSC is an example. OTSC is now being used to control gastrointestinal bleeding as well as to close gastrointestinal fistulae and acute gastrointestinal perforation [16–34]. The OTSC system is simple to use and takes minimal time; therefore, it is an appropriate choice for endoscopic wound closure. The literature contains studies of the OTSC, most of which are pilot, experimental, or animal studies; however, to date, the clinical applications of the OTSC have not been fully evaluated. In eight cases, Sarker [3] successfully used the OTSC closure system for the resection of gastrointestinal submucosal tumors (not originating from the muscularis propria) and wound closure. In the study of Fähndrich M, Sandmann M [34], a total of 17 patients underwent EFTR using the OTSC system. The technical success was 94  % (16 /17). The complete resection (R0) rate was 100  %. There were no complications.

In this study, we evaluated the safety and feasibility of the OTSC system for the closure of gastric defects after EFTR for gastric tumors originating from the muscularis propria. Our study results have shown EFTR to be effective for the resection of such tumors. As confirmed by pathology, the full-thickness resection rate of tumors was 23/23 (100 %), and no residual tumor was observed at the resection margin. This study demonstrated that iatrogenic gastric perforations were successfully closed with OTSC in all 23 cases of EFTR. Furthermore, fistula closure with OTSC significantly shortened the closure time. The average time of gastric defect closure was 4.9 min in this study.

In many previous studies, metal clips were commonly used to close iatrogenic perforations after gastric resections. Generally speaking, closing small gastrointestinal perforations with metal clips is convenient. Fujishiro et al. [35] reported 27 cases of gastrointestinal perforations at different sites after EMR that underwent closure with metal hemostatic clips. The average diameter of the defects closed was 2.5 mm (maximum: 5 mm), the average closure time was 10 min (range 8–20 min), and the average number of metal clips used was three (range 1–8). However, closing large gastric perforations with metal clips is difficult and time consuming; furthermore, it requires a high level of technical expertise. In addition, there is a certain risk of dehiscence at the closure site following wound closure with metal clips alone; this is because metal clips primarily approximate the gastric mucosa and cannot completely approximate the gastric submucosa and muscularis propria. Considering the above, based on gastric defect closure with metal clips, Ye et al. [4] evaluated the use of nylon suture snares to connect all the metal clips in order to secure their closure. Von Renteln et al. [23] compared the closure effect of conventional endoscopic clips vs. OTSC closure clips on gastric defects and found that the OTSC closure clips were significantly superior to conventional endoscopic clips in terms of the operative time (8.5 vs. 31.5 min).

In general, OTSC can rapidly and accurately close gastrointestinal perforations <20 mm [2, 16–34]. In a prospective, multicenter study, Voermans et al. [27] treated 36 cases of acute gastrointestinal perforations (including five cases of esophageal perforations, six cases of gastric perforations, 12 cases of duodenal perforations and 13 cases of colon perforations). Of the 36 cases, 33 (89 %) successfully underwent closure with the OTSC closure system and the operative time was 5.44 ± 4.15 min. In a report by Schlag et al. [28], six patients suffered iatrogenic perforations during endoscopic dissection and resection of gastric tumors; however, wound closure with OTSC was successful in all patients.

OTSC has a rapid, well-established hemostatic effect on gastrointestinal bleeding. Kato et al. [30] prospectively compared the hemostatic effects of three different endoscopic clips on gastrointestinal bleeding and found that OTSC closure clips were superior to conventional metal clips in terms of both the operative time and operative effect; the average operative time of OTSC closure clips was 49.2 s, compared with two brands of conventional metal clips, which required 284.1 and 336.8 s.

The OTSC closure system has a certain restrictive requirement in regard to the perforation size. It is difficult to close a perforation >3 cm [2, 31], slightly difficult to close a perforation >2 cm (complete closure cannot be achieved using one OTSC closure system in some cases) [2, 31] and is suitable for closure of a perforation ≤2 cm [2, 16–31].

This study has some limitations in regard to the study design; it was a single center, retrospective study based on a small sample size and a short follow-up time. This new technique must be further investigated in larger, multicenter, randomized, controlled, prospective studies.

In conclusion, the OTSC closure system can be used for gastric defect closure after EFTR for gastric tumors originating from the muscularis propria (tumor diameter ≤2 cm). It is simple to perform, convenient, safe and effective. This new technique requires further investigation.

## Electronic supplementary material

Below is the link to the electronic supplementary material.

Supplementary material 1 (MPG 326088 kb)
